# A System of Practical Surgery, with Numerous Explanatory Plates and Drawings after Nature

**Published:** 1840-10

**Authors:** 


### Art. XVI.

*A System of Practical Surgery, with numerous explanatory plates and drawings after nature.* By John Lizars, Professor of Surgery to the Royal College of Surgeons, and lately Senior Operating Surgeon to the Royal Infirmary of Edinburgh. Part II.—*Edinburgh,* 1839. 8vo, pp. 333.

In a previous Number we called the attention of our readers to the first part of Mr. Lizars's "System of Practical Surgery," and we there expressed our opinion that the book was not of the character which should belong to a work bearing this title. We are happy to see that our observations have been followed by an improvement of some of the defects which were most prominent in the first part; but we are still under the disagreeable necessity of stating that, as a system of practical surgery, the second part can be regarded as in no respect superior to the former. We do not mean to say that there is not much valuable practical matter in this volume; and if this matter had been published in a less pretending form, it would have been not merely in stricter accordance with its claims to notice, but would have been more generally useful than it can be in its present shape. It contains some of the results of Mr. Lizars's own experience, which would have made a valuable paper for the Transactions of some Medico-Chirurgical Society, or might have passed through one or more editions in the form of a little independent volume, with a title corresponding in humility to the value of its contents.

That our present judgment may not appear to have been formed hastily or carelessly, we will here set down some of the remarks that we made in reading the chapters on the diseases of the eye, the ear, and the teeth: we might easily enlarge our evidence from most of the other parts of the volume.

" *Inflammation of the Eye, or Ophthalmia.* This affection has been divided and subdivided by some authors into so many varieties, that the student is perfectly perplexed. That affecting the tissues is the most fashionable, as conjunctivitis, corneitis, sclerotitis, choroiditis, retinitis, hyalitis, capsulitis, lentitis, and aquo-capsulitis; and to render this catalogue complete, we have only to add aquitis, ciliaritis, vitreitis, uveitis, Ruyschitis, Monroitis, and Jacobitis. This catalogue I intend to reduce to four,—acute inflammation, chronic inflammation, purulent inflammation, and iritis; even the purulent might be described along with the acute ophthalmia." (p. 43.)

The account of the symptoms and treatment of acute ophthalmia which follows, is the best commentary which could be offered on the absurdity of what is here so flippantly inculcated; for sure we are, a student will never derive a single definite idea from such a jumble. If he tries to be guided by it, he will just as likely subject the patient labouring under a slight external ophthalmia to the energetic treatment necessary for an internal inflammation, and *vice versâ*, as adopt what is proper for each case. When the diagnosis is undecided, the treatment will be always, at the best, undecided.

We consider it our duty strongly to protest and warn our younger readers against the proceedings recommended in the following extract, jumbled together as they are, and founded on indications so vaguely described:

" When the conjunctiva investing the eyelids is tumid and congested, it should be touched with the sulphate of copper or nitrate of silver, applying immediately after a slip of lint dipped in olive oil, and afterwards warm water for a day or two, then the rose water, and ultimately the zinc or silver collyrium. The crude copper (?) or silver (?) ought to be repeated oftener than once in the six days. *In children, this state of the conjunctiva leads to the eversion of the eyelids, particularly the upper, and thus named lagophthalmos.* This is to be treated in the same way, or with a glass rod, having a drop of nitric acid, swept along the conjunctiva palpebræ. This surface, when thus affected and neglected, ultimately degenerates into a granular state, especially in the scrofulous, and requires the same applications; but if not cured, the eyelids should be everted with a probe, [the eyelid has just been described as unnaturally everted,] and the granular surface paired off with an iris knife, or small scalpel, such as that used in the dissecting-room for tracing the nerves. If the eyeball has a full appearance, and seems to be keeping up the irritation, the cornea should be punctured with a needle in its lower aspect, to evacuate the aqueous humour, which instantly moderates the inflammation, by removing the tension." (p. 456.)

Any young practitioner taking this for judicious and trustworthy advice, and attempting to act up to it without any qualification, would make sad havoc among the eyes of his unhappy patients. We have put one sentence in the above extract in italics, as it gives what is certainly a new definition of lagophthalmos.

Mr. Lizars informs us (p. 47) that in purulent ophthalmia both eyes are commonly involved, especially when gonorrhœal matter has been applied. In this he is at issue with Mr. Lawrence, who says (*Treatise*, p. 224,) " In general, gonorrhœal ophthalmia attacks only one eye, while the purulent disease affects both;" and Dr. Mackenzie, who says (*Practical Treatise, 2d ed.*, p. 438,) " in general, it is only one eye which is affected with this disease (gonorrhœal ophthalmia). Whenever we see one eye affected with severe puro-mucous inflammation, the lids much swollen, and of a livid colour, and the discharge copious, without any affection of the other eye, we may suspect the case to be gonorrhœal." Dr. Vetch, speaking of the Egyptian ophthalmia, says, "there is not one case in a thousand in which one eye only becomes affected."

Mr. Lizars evidently jumbles together purulent ophthalmia, ophthalmia neonatorum, and gonorrhœal ophthalmia. The purulent ophthalmia of Mr. Lizars is said by him to be "in this country (Scotland) almost always produced by the application of gonorrhœal matter to the eye" (gonorrhœal ophthalmia). The experience of Dr. Mackenzie in Glasgow does not warrant the view that purulent ophthalmia, as it is commonly understood, and ophthalmia neonatorum are in Scotland always the result of gonorrhœal inoculation.

"*Iritis.* Inflammation of the iris is almost exclusively developed in the syphilitic constitution, like purulent ophthalmia in the gonorrhœal, the difference being, that gonorrhœal matter applied to the eye of an individual otherwise free from gonorrhœa, will excite purulent inflammation, while any exciting cause, as cold winds, will produce iritis in a patient labouring under syphilis." (p. 48.)

Though it is quite true that the syphilitic is a very common form of iritis, it is wrong to say it is the almost exclusive one. This one-sided view of Mr. Lizars strangely contrasts with the contrary but still more one-sided view of Mr. Hunter and Mr. Pearson.

"Whenever pus is detected in the anterior chamber it should be evacuated; and whenever there is any projection behind the iris, the sclerotic coat should be punctured." (p. 50.)

This is certainly rash and unwarranted practice as a general rule.

Mr. Lizars not only repeats the current but erroneous notion of the pathology of staphyloma, but even affects to describe the progress of its development. Arrest the progress of *staphyloma* by the administration of Fowler's solution! This is an example of the vagueness of Mr. Lizars's pathology and therapeutics of the eye.

"Closure of the pupil. This supervenes to either common ophthalmia, syphilitic iritis, or adhesion of the iris to the cornea, which last is named syncopia. In these cases lymph is effused, which prevents the rays of light from passing through the pupil, and the forming an artificial pupil now becomes a question." (pp. 56-7.)

Such is Mr. Lizars's luminous account of closure of the pupil, and his only indication for the operation of forming an artificial pupil. In conformity with such a contracted view of the subject, the only operation he describes is that by incision. In regard to the other modes of operating, he merely says:

"There are also excision, or corectomia, or iridectomia, and separation, or coredialysis, or iridodialysis, with numerous modifications of each of the three, and innumerable instruments, which I deem it unnecessary to describe." (pp. 57-8.)

Mr. L. ought to have stated, for the benefit of his young readers, that the different modes of forming an artificial pupil are each adapted to particular morbid states of the eye, and that one cannot be always adopted instead of another.

But the operation recommended by Mr. Lizars that by incision, he has incorrectly described and figured. It is clear that he is quite ignorant of the principle of the operation by incision, when he says the operator must be careful not to wound "even the capsule of the lens." The operation in fact *cannot* be done without wounding the lens—no operator ever attempts it, so that breaking up or depressing the lens, if this is present, is in general a part of the operation. Sir W. Adams, who revived the operation, thought the cause of his success was his plan of putting a piece of the broken lens between the lips of the incision in the iris, and thus preventing reunion. The frequent failure of the operation in the hands of others he attributed to the neglect of such a precaution. But Sir W. Adams was wrong. The operation only succeeds in those cases in which the iris still retains its natural texture, and is capable of contracting, so that when cut the incision gapes open and continues so.

When the operator has reason to suppose that both lens and capsule are transparent he will choose another mode of operating.

Mr. Lizars's figure (plate viii. fig. 10,) gives quite a wrong idea of the operation, and is altogether unlike that of Cheselden in the Philosophical Transactions.

The operation of making an artificial pupil by incision of the iris cannot be said to be "unquestionably the best," as Mr. Lizars affirms it is. The cases adapted for it (cases in which the natural structure of the iris has not been changed by inflammation), not having been always properly known, the operation has failed so often that the German surgeons appear to have discarded it altogether—speaking of it in their works merely in an historical point of view—though certainly unjustly. In *cases adapted for it* we consider it unquestionably the best and simplest operation, provided the surgeon knows the principle of it and knows how to perform it—not attempting to do what cannot be done, i. e., to avoid wounding "even the capsule of the lens."

"*Operation of Extraction of the Lens.*" Having made the section of the cornea, the operator, says Mr. L. (p. 63), "then elevates the cornea with a pair of ordinary dissecting forceps, and next scratches the capsule with a needle or curette," &c. To elevate the cornea with a pair of ordinary dissecting forceps is surely most rude surgery. What necessity is there for elevating the cornea at all? The instrument for lacerating the capsule can be readily enough insinuated under the flap of the cornea.

The case of hydatid in the anterior chamber referred to by Mr. Lizars, p. 73, (and which in his careless manner he terms cysticercus cellulosus, and classes among the entozoa,) appears to be the same as that described and figured by Mackenzie. Mr. Lizars's figure of the eye (plate viii. fig. 8,) is not very intelligible; it certainly was not drawn from nature.

"The cornea is sometimes too convex, named *myopia*, at others too flat, termed *presbyopia;* hence there are short-sighted and far-sighted people, who require different shaped lenses to remedy the defect. One eye, however, has been alone thus affected [how affected—the cornea too convex or too flat?] and increased until it has become prominently conical," &c. (p. 74.)

Surely Mr. Lizars does not mean that any slight increase of convexity which may exist in a myopic eye is an early stage of conical cornea.

The following is an instance of the disregard of accurate language which we had occasion to reprehend in Mr. Lizars's former volume, and which we regret to see still so prevalent in the present: "The iris is sometimes deficient, while in *others* [?] there is a rudimental process, generally at the inferior margin of the ciliary processes; all of *whom* [the ciliary processes?] have indifferent vision, some being nearly blind." (p. 75.)

We shall not proceed further with our notice of this chapter on the Diseases of the Eye; it is, however, but just to remark that the part on the diseases of the lachrymal organs is better than the rest.

If we were to extend our remarks to some other chapters in the work, we fear we must adopt the same strain of criticism, however disagreeable it might be to us to do so. Thus in the chapter on Diseases of the Ear we find the individual subjects in no respect more clearly or scientifically discussed. A student reading this chapter would, in fact, be led to suppose that, instead of the diagnosis of the diseases of the ear being somewhat obscure, and the diseases themselves not always very amenable to treatment, the former always is readily to be made, and the cure forthwith accomplished.

In the chapter on the Diseases of the Teeth, again, we have an equally marked specimen of the flippant dogmatism, careless language, and indefinite views which unfortunately characterize, more or less, the whole work. Indeed, we are once more compelled to say that few parts of the volume indicate on the part of the writer that precision of ideas on the subjects treated of which alone qualifies for the difficult task of efficiently communicating instruction to others. We have, however, no doubt but that Mr. Lizars wields his surgical instruments to much more effect than he does his pen, and we are sure his patients derive more advantage from the former than his readers are likely to do from the latter.

We shall conclude our notice of Mr. Lizars's "System" with the following quotation from a quaint old book—*The Art of Healing*, by Thomas Marryat, M.D.:

"It is a trite but just observation, that those who are most dogmatical are most apt to be erroneous. Self-importance has a mighty bad effect on the eyes, and wonderfully impairs their visual power. Of this the author begs leave to deliver the following instance; in the year 1756, having made (in his own opinion) a momentous discovery, he gave it to the public with a most imperious and dictatorial air of self-consequence, under the title of Medical Aphorisms. The authors of the Monthly Review, piqued to see the public so cavalierly treated, applied a critical vesicatory with such propriety, as entirely to evacuate the peccant humour, and (beyond their own expectations) to effectuate a perfect cure. Would to Heaven that every author who falls under their castigatory pen would make the same use that he did of the well-meant hints of those ingenious and never-enough-to-be-applauded writers, instead of snarling at the rod, or impotently snapping at the kind hand that holds it."

